# Progress in Medicine

**Published:** 1900-05-19

**Authors:** 


### Progress in Medicine.

### DISEASES OF THE DIGESTIVE SYSTEM.

(*Continued from page* 33.)

The differentiation of chronic asthenic gastritis from chronic sthenic gastritis can only be made by analysis of the stomach contents. In the former there is depression of the glandular function, in the latter an excess of hydrochloric acid. Boardman[10] gives the following directions for the treatment of asthenic cases. The diet must contain no cane sugar, and be as free as possible from yeast bread, rolls, malt liquors, and vegetables containing tough, hard cellulose. Small quantities of vegetables may be given in pulse form, cocoa substituted for tea or coffee, and not more than half a pint of fluid taken with meals. Lavage of the stomach is useful, and in severe cases a silver nitrate solution (five grains to the pint) may be used. Outdoor exercise is very beneficial. Laxatives are generally needed. Hydrochloric acid and pepsin are useful, and, as adjuncts, carbolic acid or resorcin. Chronic sthenic gastritis (acid gastric catarrh) should be dealt with by avoiding mental worry and sexual excess. The food should be fully masticated, and whilst consisting largely of proteid, may contain carbohydrate which is rendered harmless by the addition of diastasic preparations. Severe cases do well on milk, eggs, and gluten preparations. Acid fruits are injurious, but baked apples, white grapes, and bananas are easily tolerated. A glass of plain water drunk every half-hour during digestion will often do good. Wines, spirits, and condiments are injurious. Lavage with alkaline solutions should be systematically performed, and intragastric electricity is of great value. For neutralising the excessive acidity the following mixture is recommended: Magnes. Ust., ʒi.-ʒiv.; Cret. prepar., ʒss.-ʒii.; Bism. subnit.. ʒi.-ʒii.—M. and ft. chart xii. The excessive secretion of hydrochloric acid may sometimes be controlled by belladonna or nitrate of silver.

Goodhart[11] deprecates the routine treatment of acidity, and the uric acid diathesis. The cause of this form of dyspepsia may be visceral sluggishness, anxiety, shock, nervous exhaustion, hereditary gout, or kidney disease. Each case must be considered separately, and many will be found to benefit by articles of diet generally forbidden, such as meat, sugar, fats, beer, and wine.

Schwarz[12] maintains that in cirrhosis of the liver with digestive disturbance, neither nitrogenous nor amylaceous foods need be withdrawn, provided the diet be highly nutritious, in small quantity, and leave little residuum. Fat must be withheld if there be jaundice, and abstinence from alcohol, tea, and coffee enjoined. A rigid milk diet is safest if there be serious hepatic symptoms.

**The Liver.**—Abbott[13] gives particulars of the clinical history of the bacteriological investigations into a case of progressive portal cirrhosis due to the colon bacillus. Many varieties of this bacillus have been described, and its morphological variability is well recognised. In the case under notice the organism was found as a diplococcus in the liver cells, and in the ascitic fluid, and when cultivated on ascitic fluid retained its diplococcoid form. After several passages through guinea pigs, the coccus took on the typical colon characteristics, and a similar change could be brought about by repeated and prolonged sub-cultures on various media. These observations are of extreme interest in connection with the theory of the infective nature of cirrhosis of the liver, a theory supported chiefly by the French school and by Adami.

A comparison between the different varieties of hepatic cirrhosis is given by Cheadle.[14] It is doubtful whether a strict line can be drawn between atrophic and hypertrophic cases, or whether the enlargement and diminution of the liver may not be stages of the same disease. The distinctive symptoms of atrophic disease are ascites, perihepatitis, and associated Bright's disease. In hypertrophic cases ascites is rarer, jaundice more common. Atrophic changes are multilobular, and generally excited by alcohol. Syphilis is the commonest cause of the hypertrophic disease. With fibrosis of the liver is often associated fibrosis of other organs, degeneration of cardiac muscle, and liability to erysipelas and tuberculosis. With regard to prognosis, the more favourable cases are those in which the liver is enlarged, the sufferer of a not too advanced age and in a fair state of nutrition. Ascites is always a bad sign, but patients may live many years even after extreme ascites necessitating repeated tappings. In the treatment of cirrhosis, all alcohol, stimulating food, fats, and sugar should be reduced to a minimum. Mild laxatives are needed, and, in suitable cases, mercury, potassium iodide, and digitalis are invaluable. Ascites is not infrequently in part due to accompanying debility and dilatation of the heart, and is best met by early and repeated paracentesis.

Osler[15] records two cases of hypertrophic cirrhosis of the liver with bronzing of the skin—hæmochromatosis. These cases are characterised by a widespread deposition of iron-containing pigment in certain cells, and by the formation of iron-free pigment in normally pigmented areas. The deposition of the pigment in the liver and pancreas leads to necrosis of the cells, inflammatory changes, and hypertrophy. From the pancreatitis diabetes ensues, and generally ends fatally. So-called bronzed diabetes occurs exclusively in men. A large quantity of sugar may be passed, and on post-mortem examination the pancreas is found deeply pigmented and cirrhotic.

**Hepatic Tuberculosis.**—Three varieties of this disease are described by Moore[16]—miliary tuberculosis, solitary tubercle of the liver, and diffuse interstitial hepatitis. Miliary tuberculosis is the commonest form, and the path of infection is the hepatic artery, or the umbilical vessels in congenital cases. It generally occurs in old standing cases of phthisis, in tuberculous peritonitis, or general tuberculosis. Fraenkel states that the signs of tuberculous invasion of the liver are early jaundice, respiratory disturbances, and the typhoid state. Solitary tubercle may appear either as caseating cavities in connection with the bile ducts, or as detached central caseating masses. They occur in association with tuberculous ulceration of the intestine, and are very rare. The author gives details of a case of cancer of the pylorus associated with conglomerate tuberculosis of the liver, spleen, and cœliac glands. Tuberculous hepatitis is not infrequently met with in experimental tuberculosis of animals. It is met with most frequently in children, and is associated with retrograde changes and fatty infiltration from the liver cells.

**Floating Liver.**—According to Einhorn[17] floating liver is a not uncommon disease, especially in women. The causes are laceration of ligaments, frequent forcible expirations, and progressive emaciation. Cases may present one or other of the following groups of symptoms—(1) Dyspepsia, weakness, and nervousness; (2) hepatalgia, pains of a drawing and tearing character; (3) hepatic colic without jaundice; (4) asthmatic attacks, dyspnœa with fulness and constriction in the upper abdominal region. The upper border of the liver in the mammary line may reach the seventh rib or costal margin, and the organ may undergo anteversion, retroversion, lateral, vertical, or oblique displacement. Complete or partial reposition should be possible. The disease is not unfrequently associated with floating kidney. The condition can generally be improved by hygienic measures, massage, hydrotherapy, and a liberal diet. Operative interference has not met with marked success.

10 Int. Med. Mag., Dec., 1899, et seq. 11 Lancet, Jan. 6, 1900. 12 Centrab. f. d. ges. Therap., Aug., 1899. 13 Jour. Pathol. and Bac., Feb., 1900. 14 Brit. Med. Jour., Mar. 31, 1900, et seq. 15 Ibid, Dec. 9, 1899. 15 Med. Chron., Oct., 1899. 17 Med. Rec., Sept. 16, 1899.

